# Evaluating indwelling devices and other risk factors for mortality in invasive Carbapenem-resistant Enterobacterales infections in Georgia, 2012–2019

**DOI:** 10.1017/ash.2023.531

**Published:** 2024-01-02

**Authors:** Lucy S. Witt, Mary Elizabeth Sexton, Gillian Smith, Monica Farley, Jesse T. Jacob

**Affiliations:** 1 Division of Infectious Diseases, Emory University School of Medicine, Atlanta, GA, USA; 2 Georgia Emerging Infections Program, Atlanta, GA, USA; 3 Atlanta Veterans Affairs Medical Center, Atlanta, GA, USA

## Abstract

**Objective:**

Carbapenem-resistant Enterobacterales (CRE) infections are a public health threat due to the risk of transmission between patients and high associated mortality. We sought to identify risk factors for mortality in patients with invasive CRE infections and to specifically evaluate whether there was an association between indwelling medical devices and 90-day mortality.

**Design:**

Retrospective observational cohort study of patients infected with CRE in the eight-county metropolitan Atlanta area between 2012 and 2019.

**Methods:**

Patients with invasive CRE infections were identified via the Georgia Emerging Infections Program’s active, population- and laboratory-based surveillance system and linked with the Georgia Vital Statistics database. We used bivariate analysis to identify risk factors for mortality and completed log binomial multivariable regression to estimate risk ratios (RR) for the association between indwelling devices and mortality.

**Results:**

In total, 154 invasive CRE infections were identified, with indwelling devices present in most patients (87.7%) around the time of infection. Admission to an intensive care unit was found to be associated with 90-day mortality (adjusted RR [aRR] 1.55, 95% CI 1.07, 2.24); however, the presence of any indwelling device was not associated with increased risk of 90-day mortality in multivariable analysis (aRR 1.22, 95% CI 0.55, 2.73). Having at least two indwelling devices was associated with increased mortality (aRR 1.79, 95% CI 1.05, 3.05).

**Conclusions:**

Indwelling devices were prevalent in our cohort but were not consistently associated with an increased risk of mortality. Further studies are needed to examine this relationship and the role of device removal.

## Introduction

Carbapenem-resistant Enterobacterales (CRE) infections are a rapidly growing public health emergency.^1^ In 2019, there were approximately 6,000 cases of CRE in the United States (US), and rates have risen since the onset of the COVID-19 pandemic.^2^ CRE’s threat stems both from a high associated mortality (as compared to infections with carbapenem-susceptible organisms^3–5^) and from the potential transmissibility of resistance genes to other organisms, as 30%–80% of CRE organisms harbor carbapenem resistance genes, many of which reside on mobile plasmids.^1,6–9^

Enterobacterales can be a part of the normal human microbiome in the gastrointestinal and genitourinary tracts. As such, CRE may be nonpathogenic in non-sterile body sites such as wounds, respiratory secretions, and urine. Prior studies characterizing the rates of CRE and demographics of afflicted patients have included colonization, which may have limited their ability to identify risk factors for mortality.^10–12^ Understanding the characteristics of patients with invasive CRE infections and their risk factors for mortality provides a stronger analysis and may suggest future interventions.

Indwelling medical devices including urinary catheters, central venous catheters (CVCs), and endotracheal tubes are common in the modern US healthcare system and are frequently encountered in critically ill and healthcare-exposed patients. Prior studies have suggested that indwelling devices may be a risk factor for acquiring CRE as well as for progression from CRE colonization to invasive disease.^10,13^ They have not, however, been consistently linked to mortality risk in patients with CRE.^14^ Overuse of indwelling devices is known to have multiple negative impacts, and these devices could represent a modifiable risk factor for mortality in patients with CRE infections, given the potential to avoid or reduce their use in high-risk patients.

We performed a retrospective observational cohort study to describe sterile site CRE infections and identify associated risk factors for mortality in the Atlanta metropolitan area between 2012 and 2019. We evaluated the annual incidence of infection, as well as the clinical characteristics of patients affected. Using this data, we identified risk factors for all-cause 90-day mortality in patients with invasive CRE infections and developed a model to assess the risk of mortality specifically associated with indwelling devices, a potentially modifiable risk factor.

## Methods

### Study population

This was a retrospective observational cohort study utilizing data obtained by the Georgia Emerging Infections Program (EIP) (funded by the Centers for Disease Control and Prevention [CDC]) from 2012 to 2019. The Georgia EIP performs active population- and laboratory-based surveillance for CRE in the eight counties surrounding metropolitan Atlanta (Supplementary Figure 1). EIP staff routinely query laboratory testing instruments at each associated microbiology laboratory and identify all relevant organisms that are carbapenem-resistant including *Escherichia coli*, *Klebsiella* spp., and *Enterobacter* spp. Only patients residing in the catchment area are eligible for inclusion in the dataset. For this study, we included only specimens from normally sterile sites, such as blood or peritoneal fluid (Supplementary Table 1), using the first isolate of CRE from each patient in a 30-day period. One patient contributed twice to the dataset having two incident infections, five months apart. This study was approved by the Emory University Institutional Review Board.

### Data collection

EIP staff perform chart review of identified cases to capture data in a standard 29-item case report form, including basic patient demographic information such as race and ethnicity, infection site, risk factors including the presence of indwelling devices and patient comorbidities, outcomes including length of stay, admission to an intensive care unit (ICU), and mortality, and isolate antibiotic susceptibilities. Indwelling medical devices were defined as a CVC, urinary catheter, or any other indwelling medical device in place at any time in the two calendar days prior to infection (Supplementary Table 2). Patient comorbidities were evaluated using a Charlson comorbidity index (CCI) score, which was dichotomized to those with scores less than or equal to two and those with scores higher than two. Length of stay was calculated from admission and discharge date. ICU admission was defined as the patient being in the ICU at any time in the seven days prior to isolate collection. Ninety-day, all-cause mortality after infection was obtained by linking the Georgia EIP patient registry with the Georgia State Office of Vital Records.

### Carbapenem resistance

In 2012, the EIP definition for CRE required non-susceptibility to meropenem, doripenem, or imipenem (MIC ≥ 4 μg/mL) with resistance to all tested third-generation cephalosporins. However, in 2016, the definition was simplified to include any relevant organism resistant to meropenem, doripenem, imipenem, or ertapenem (MIC ≥ 2 μg/mL) and susceptibility breakpoints were updated to better align with state public health definitions. To avoid confounding due to this definition change, only patients meeting the 2012 definition across the full-time period (2012–2019) were included in this study (Supplementary Table 3).^10^

### Data analysis

Trends over time were assessed using a longitudinal analytic approach including generalized estimating equations to evaluate rates of infection per population. Descriptive univariable analyses of basic patient demographics were completed for the entire cohort. Bivariate analyses of continuous and categorial variables’ association with 90-day mortality were conducted using Wilcoxon rank sum or chi-squared test (or Fisher’s exact test as appropriate). Patients never admitted to the hospital and those with missing data regarding hospitalization were excluded from analyses involving hospital length of stay. An investigator created directed acyclic graph (DAG) based on authors’ knowledge of CRE and a nonsystematic literature review was used to inform variable inclusion in the multivariable analysis (Supplementary Figure 2). Multivariable log binomial regression was used to estimate risk ratios (RR) for the association of CCI >2, ICU admission, long-term acute care hospital (LTACH) stay in the last year, and presence of an indwelling medical device with 90-day mortality. Secondary analyses were completed evaluating whether there was an association between presence of a CVC and mortality, as a CVC was the most common indwelling device and may pose a unique risk for invasive infection, and whether there was an association with presence of at least two indwelling medical devices and mortality. ICU admission was not included in the multivariable model for this secondary analysis due to concern for collinearity between multiple devices and ICU admission, but a sensitivity analysis was completed that included ICU admission to assess its effect on the relationship and the assumption of collinearity. Chronic dialysis was not included in any multivariable model due to collinearity with possession of an indwelling device, although the association between indwelling medical devices and 90-day mortality was stratified by chronic dialysis to assess for interaction. A two-sided *p*-value of 0.05 was considered significant, and all analyses were completed using SAS 9.4 (SAS Institute, Cary, NC).

## Results

### Demographic characteristics and trends over time

**Figure 1.**
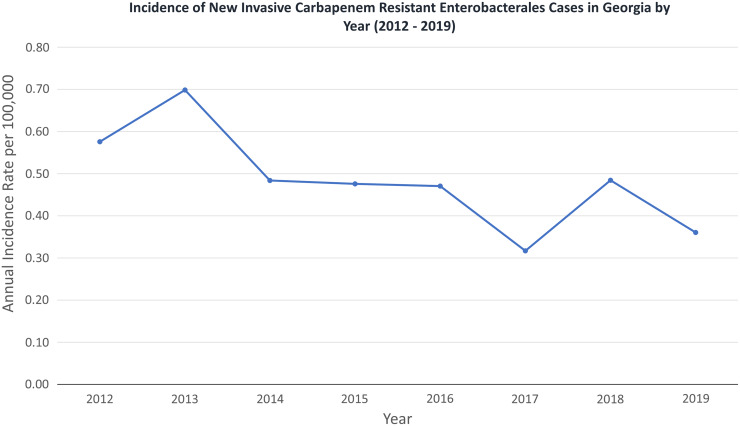
Rates of carbapenem-resistant Enterobacterales infections in Georgia 2012–2019.

There were 154 incident cases of invasive CRE infection in the Atlanta area between 2012 and 2019. A majority of patients with invasive CRE infections were male (53.2%); 88.3% were admitted to the hospital at the time of infection, for a median of 20 days (Table 1). Most infections occurred in the bloodstream (84.4%) and 65.6% were *Klebsiella pneumoniae* (Table 1). Indwelling devices were frequent (87.7%) with the most common device being a CVC (70.8%). One-fifth of patients had both a tracheostomy tube and a percutaneous enteric gastronomy (PEG) tube in place, and 78% of that group had a concurrent CVC. A majority (53.6%) of patients were admitted to the ICU at any time during their hospitalization, and 31.2% had been in the ICU in the seven days prior to isolate collection. Most patients had been admitted to a hospital (81.2%) and 24% had stayed in a LTACH in the last year (Table 1). In-hospital mortality was 23.4% for the entire cohort and 90-day mortality was 42.2%. The annual incidence rate of invasive CRE infections per 100,000 persons decreased over the study period from 0.576 to 0.361 (Figure 1).

**Table 1.** Characteristics of patients with invasive carbapenem-resistant Enterobacterales infections by those with and without indwelling medical devices, Atlanta, Georgia, 2012–2019

	Total *N* =154 *n* (%)	Without Indwelling Device *N* = 19 *n* (%)	With Indwelling Device *N* = 135 *n* (%)
Age, median (IQR)	59 (48–69)	54 (33–67)	61 (48–69)
Female	72 (46.8)	11 (57.9)	61 (45.2)
Race* ^¥^			
Black	103 (66.9)	12 (63.2)	91 (67.4)
White	40 (26.0)	6 (31.6)	34 (25.2)
Pacific Islander	5 (3.3)	0 (0)	5 (3.7)
Ethnicity* ^¥^			
Hispanic	4 (2.6)	0 (0)	4 (3.0)
Non-Hispanic	122 (79.2)	16 (84.2)	106 (78.5)
Charlson comorbidity index score >2*	61 (39.6)	4 (22.2)	57 (42.5)
Length of stay, median (IQR)**	20 (10–39)	16 (5.5–23.5)	21 (10–40)
Intensive care unit admission*	48 (31.2)	2 (10.5%)	46 (34.1%)
Immunocompromised ^¥^	36 (23.4)	5 (26.3)	30 (22.2)
HIV or AIDS	5 (3.3)	1 (5.3)	4 (3.0)
Transplant (bone marrow)	1 (0.7)	0 (0)	1 (0.8)
Transplant (solid organ)	2 (1.3)	0 (0)	2 (1.5)
Solid tumor	15 (9.7)	1 (5.3)	14 (10.4)
Metastatic cancer	10 (6.5)	2 (10.5)	8 (6.0)
Hematologic malignancy	5 (3.3)	1 (5.3)	4 (3.0)
Specimen Source			
Blood	130 (84.4)	14 (73.7)	116 (85.9)
Other	24 (15.6)	5 (26.3)	19 (14.1)
Organism^¥^			
*Escherichia coli*	16 (10.4)	4 (21.1)	12 (8.9)
*Enterobacter cloacae*	19 (12.3)	6 (31.6)	13 (9.6)
*Klebsiella aerogenes*	10 (6.5)	0 (0)	10 (7.4)
*Klebsiella pneumoniae*	101 (65.6)	7 (36.8)	94 (69.6)
*Klebsiella oxytoca*	8 (5.2)	2 (10.5)	6 (4.4)
Polymicrobial infection*	58 (37.9)	6 (31.6)	52 (38.8)
History			
Previous stay in hospital (1 year)	125 (81.2)	13 (68.4)	112 (83.0)
Previous stay in long-term care facility (1 year)	57 (37.0)	2 (10.5)	55 (40.7)
Previous stay in long-term acute care (1 year)	37 (24.0)	0 (0)	37 (27.4)
Surgery	75 (48.7)	6 (31.6)	69 (51.1)
Chronic dialysis	53 (34.4)	4 (21.1)	49 (36.3)
*Hemodialysis (% of dialysis)*	*51 (96.2)*		
*Central or hemodialysis line*	*39 (76.4)*		
*Aorto-venous fistula or graft*	*12 (23.5)*		
*Peritoneal (% of dialysis)*	*2 (3.8)*		
Indwelling Devices			
Any	135 (87.7)		
Central venous catheter	109 (70.8)		
Urinary catheter	80 (52.0)		
PEG	53 (34.4)		
Tracheostomy	51 (33.1)		
Both tracheostomy and PEG	32 (20.8)		
Endotracheal/Nasotracheal tube	11 (7.1)		
Indwelling Devices—Numerical			
None	19 (12.3)		
One	29 (18.8)		
Two	58 (37.7)		
Three	48 (31.2)		
At least two devices	106 (68.8)		

Note. IQR, interquartile range; PEG, percutaneous enteric gastronomy.

*Missing Data – Race 6, Ethnicity 28, Charlson comorbidity index score 2, Length of Stay 2, ICU 3, Polymicrobial 1.

*Did not include 16 patients who were never admitted to the hospital and 2 with missing data.

Other site of infection: bone (1), deep tissue (1), joint/synovial (1), liver (2), ovary (1), peritoneal fluid (12), pleural fluid (2), other normally sterile site (4).

All continuous variables were analyzed using Wilcoxon rank sum. All categorical variables were analyzed using chi-square except those marked with ^**¥**^ which were analyzed using fisher’s exact test.

### Patient risk factors associated with mortality

Factors statistically associated with 90-day mortality on bivariate analysis included older age, CCI score >2, admission to the ICU in seven days prior to infection, bloodstream infection, having at least two indwelling devices, and being on chronic dialysis (Table 2). Each additional indwelling medical device above one provided an additional 53% increased odds of mortality (OR 1.53, 95% Confidence Interval [CI] 1.09–2.17). In unadjusted models, ICU admission was associated with mortality with a risk ratio of 1.73 (95% CI 1.22–2.45), as was CCI score >2 (RR 1.49, 95% CI 1.03–2.15) (Table 3). Having an indwelling device present (RR 1.69, 95% CI 0.78–3.67) and previous stay at an LTACH (RR 1.31, 95% CI 0.89–1.92) were not associated with 90-day mortality in unadjusted models. In a multivariable model adjusting for ICU admission, CCI score >2, admission to LTACH, and possession of an indwelling medical device, admission to the ICU remained associated with mortality (aRR 1.55, 95% CI 1.07–2.24) while CCI score >2 (aRR 1.62, 95% CI 0.68–3.83), previous stay at LTACH (aRR 1.39, 95% CI 0.60–3.20), and having an indwelling device present (aRR 1.22, 0.55–2.73) were not (Table 3).

**Table 2.** Characteristics as related to 90-day mortality in patients with invasive carbapenem-resistant Enterobacterales infections, Atlanta, Georgia, 2012–2019

	Survived *n* = 89 (57.8%) *n* (%)	Deceased *n* = 65 (42.2%) *n* (%)	*p*-value
Age, mean (IQR)	58 (44–69)	61 (53–68)	*<0.01*
Female	42 (47.2)	30 (46.2)	0.9
Race *			0.52
Black	61 (68.5)	42 (64.6)	
White	24 (27.0)	16 (24.6)	
Pacific Islander	2 (2.3)	3 (4.6)	
Ethnicity *			0.55
Hispanic	3 (3.4)	1 (1.5)	
Non-Hispanic	68 (76.4)	54 (83.1)	
Charlson comorbidity index score >2*	29 (33.0)	32 (50.0)	*0.03*
Length of stay, median (IQR)*	20 (10–36)	20 (8–40)	0.21
Intensive care unit admission*	19 (21.4)	29 (44.6)	*<0.01*
Specimen Source			0.16
Blood	72 (80.9)	58 (89.2)	
Other	17 (19.1)	7 (10.8)	
Immunocompromised	18 (20.2)	18 (27.7)	0.28
Organism			0.27
*Escherichia coli*	13 (14.6)	3 (4.6)	
*Enterobacter cloacae*	10 (11.2)	9 (13.9)	
*Klebsiella aerogenes*	7 (7.9)	3 (4.6)	
*Klebsiella pneumoniae*	55 (61.8)	46 (70.8)	0.34
*Klebsiella oxytoca*	4 (4.5)	4 (6.2)	
Polymicrobial infection*	33 (37.5)	25 (38.5)	0.9
Indwelling Devices			
Any	75 (84.3)	60 (92.3)	0.13
Urinary catheter	44 (48.9)	36 (40.0)	*0.04*
Central line	60 (67.4)	49 (75.4)	0.28
≥ Two devices	53 (59.6)	53 (81.5)	*<0.01*
Number of indwelling devices	2 (1–3)	2 (2–3)	*0.02*
History			
Previous stay in hospital (1 year)	71 (79.8)	54 (83.1)	0.61
Previous stay in long-term care facility (1 year)	31 (34.8)	26 (40.0)	0.51
Previous stay in long-term acute care (1 year)	18 (20.2)	19 (29.2)	0.2
Surgery	50 (56.2)	25 (38.5)	*0.03*
Chronic dialysis	23 (25.8)	30 (46.2)	*<0.01*

Note. IQR, interquartile range.

*Missing data: Race 6, ethnicity 28, Charlson comorbidity index 2, length of stay 2, ICU 3, polymicrobial 1.

Length of stay data did not include 16 patients not admitted to the hospital

**Table 3.** Risk ratios for 90-day mortality in patients with invasive carbapenem-resistant Enterobacterales infections, Atlanta, Georgia, 2012–2019

	Unadjusted	Adjusted 1	Adjusted 2	Adjusted 3
	RR (95% CI)	RR (95% CI)	RR (95% CI)	RR (95% CI)
Indwelling device present	1.69 (0.78, 3.67)	**1.22 (0.55, 2.73)**		
Central venous catheter present	1.26 (0.81, 1.97)		**1.06 (0.67, 1.68)**	
>/= 2 Indwelling devices	2.00 (1.18, 3.38)			**1.79 (1.05, 3.05)**
Charlson comorbidity index score >2	1.49 (1.03, 2.15)	1.62 (0.68, 3.83)	1.33 (0.92. 1.92)	1.36 (0.95, 1.96)
Intensive care unit admission	1.73 (1.22, 2.45)	1.55 (1.07, 2.24)	1.57 (1.08, 2.28)	
Previous stay in LTACH (1 year)	1.31 (0.89, 1.92)	1.39 (0.60, 3.20)	1.13 (0.77, 1.65)	1.14 (0.79, 1.66)

Note. RR, risk ratio; CI, confidence interval; LTACH, long-term acute care hospital.

1. Adjusted for indwelling device, Charlson comorbidity score, intensive care admission, previous stay at long term acute care.

2. Secondary analysis—adjusted for central venous catheter, Charlson comorbidity score, intensive care admission, and previous stay at long-term acute care.

3. Secondary analysis—adjusted for at least two indwelling devices, Charlson comorbidity score, and previous stay at long-term acute care.

### Secondary analyses to further explore the impact of indwelling devices

Looking solely at possession of a CVC (*n* = 109), there was no increased risk of 90-day mortality for patients in the unadjusted (RR 1.26, 95% CI 0.81–1.97) or adjusted model (aRR 1.06, 95% CI 0.67–1.68) (Table 3).

Of the 106 patients who had at least two indwelling medical devices, 86% had CVCs and 72% had urinary catheters. An unadjusted model showed an association between having at least two indwelling medical devices and 90-day mortality (RR 2.00, 95% CI 1.18–3.38) (Table 3). In the adjusted model without ICU admission included, this relationship persisted (aRR 1.79, 95% CI 1.05–3.05). A sensitivity analysis that included ICU admission in this multivariable model showed a diminished effect estimate with a wider confidence interval for the risk of mortality from at least two indwelling devices (aRR of 1.59, 95% CI 0.93–2.77).

### Chronic dialysis and interaction analysis

Approximately one-third of the cohort was on chronic dialysis (n=54). Patients on chronic dialysis were older, with a median age of 61 (IQR 54–69) years, and more likely to be identified as Black race (79.3%) as compared to the total cohort. Almost all patients on chronic dialysis (92.5%) had an indwelling medical device. When stratified by dialysis, the odds ratio for all-cause 90-day mortality in patients with indwelling medical devices was 4.35 (95% CI 0.42–44.88) for patients on chronic dialysis and 1.55 (95% CI 0.46–5.28) for patients not on chronic dialysis.

## Discussion

In patients with invasive CRE infections in Georgia between 2012 and 2019, risk factors associated with all-cause 90-day mortality in multivariable modeling included admission to the ICU in the prior seven days and having at least two indwelling devices. Our mortality rate, 42.2%, was slightly higher than previously reported national and local rates, likely from limiting our analysis to only sterile site infections.^6,11^ The rates of invasive CRE infection decreased during our study period, which is reflected in national data.^15^ Potential mechanisms for the decrease in rates of CRE include increased focus on antimicrobial stewardship and improved infection control practices from national campaigns.^16^ Unfortunately, this trend was reversed after our study period in the wake of the COVID-19 pandemic, highlighting the need for renewed focus on infection prevention efforts.^2^

Possession of an indwelling medical device was prevalent in our cohort but was not associated with an increased risk of 90-day mortality in multivariable analysis, although having at least two medical devices was. Prior studies exploring the association between indwelling devices and mortality in the setting of invasive infections have had mixed results, although they did not focus specifically on CRE. CVCs have been shown to be associated with lower mortality in *Staphylococcus aureus* infections when not accompanied by endocarditis,^17–19^ potentially because they are a removable source of infection. Other studies have suggested that CVCs may be a risk factor for mortality in patients with drug-resistant Gram-negative bacteremia, but these studies did not exclusively evaluate CRE and focused on catheter-associated bloodstream infections, which we did not assess as a separate infection category.^20,21^ It is possible that indwelling devices are a risk factor for acquiring invasive infection with CRE but are not specifically a mortality risk. For example, Howard-Anderson et al. used Georgia EIP data to show that patients with CRE bacteriuria who had urinary catheters were more likely to develop CRE bacteremia, but they did not examine mortality.^10^ Mortality risk appears to be related to overall illness severity, which may explain the association between having at least two indwelling devices and mortality as multiple devices may simply be a surrogate marker for illness severity. However, multiple devices may also increase inflammation and/or increase the bioburden of healthcare-derived bacteria on patients’ bodies, increasing their risk of death. Therefore, it is unclear whether removing indwelling devices would improve survival in patients with invasive CRE infections. Evaluating device removal as a preventive intervention, especially in patients with multiple devices, merits further study.

Other risk variables associated with mortality, specifically ICU admission, are consistent with the hypothesis that illness severity is an important risk factor and with those previously described in the literature. Babiker et al described carbapenem-resistant Gram-negative infections, including both CRE and *Pseudomonas and Acinetobacter* spp., over almost 20 years at a single center in Pennsylvania and found an association between 30-day mortality and chronic renal disease, ICU admission, chronic liver disease, and bloodstream infection.^12^ Three additional retrospective cohort studies examining mortality risks for patients with CRE infections found that ICU admission was associated with mortality.^22–24^ While ICU admission is not a risk factor that is easily intervened upon, future studies could focus on risk factors for requiring ICU admission in the setting of an invasive CRE infection, to evaluate potential preventive interventions earlier in a patient’s course.

Patients with invasive CRE infections in Georgia between 2012 and 2019 were severely ill and highly healthcare experienced. This reflects the well-described pathogenesis of CRE, which involves exposure to multiple antibiotics and healthcare facilities where multidrug-resistant pathogens are known to reside on surfaces, the hands of personnel, and in patient rooms.^5,8,25,26^

### Limitations

This study has several limitations related to the use of retrospective chart review. First, the directionality of the relationship between an indwelling device and mortality is difficult to ascertain. Our data describes indwelling devices in place in the two days prior to culture of CRE; therefore, we cannot elucidate whether the device was placed prior to CRE infection leading to infection and death or was required due to abnormal physiology that developed in the setting of CRE infection. Our study could suggest that limiting the use of invasive medical devices may decrease mortality; however, having numerous medical devices may simply be a surrogate for illness severity. Our small sample size, compounded by the high proportion of patients with indwelling medical devices, limits our ability to observe differences between groups. Additionally, although our investigation is unique in its description of invasive CRE infections, it only represents one urban, southern US region; therefore, the risk factors identified through our study may not be generalizable to other geographic areas. Prior studies have suggested that appropriate antibiotic therapy contributes to survival in patients with CRE infections, but information regarding antibiotic therapy was not available in our dataset, thus this risk factor could not be included in our analysis. Our analysis was limited to *Escherichia coli*, *Klebsiella* spp., and *Enterobacter* spp. and did not include isolates with resistance to ertapenem alone which likely reduced confounding but may have mildly reduced generalizability. Lastly, our dataset did not include information regarding subsequent removal of indwelling medical devices and thus cannot report whether subsequent device removal affected mortality.

Our study describes invasive CRE infections in the Atlanta metropolitan area between 2012 and 2019 and identified risk factors for mortality. It failed to show a consistent association between indwelling medical devices and increased all-cause 90-day mortality, but did suggest that illness severity, particularly the need for ICU admission, was associated with mortality. Further work should be undertaken to replicate these findings within a national cohort, to explore trends in case rates and mortality after this time interval, and to evaluate potential interventions to decrease patient mortality risk.

## Supporting information

Witt et al. supplementary material Witt et al. supplementary material

## Supplementary material

For supplementary material accompanying this paper visit http://doi.org/10.1017/ash.2023.531. click here to view supplementary material

## Previous presentation

Preliminary data from this analysis was presented in poster form at the Southeast Regional Clinical and Translational Science Conference in March 2022 and at ID Week 2022 in October 2022.
