# Supplementary material for: Evaluating indwelling devices and other risk factors for mortality in invasive Carbapenem-resistant Enterobacterales infections in Georgia, 2012–2019
Source: Antimicrob Steward Healthc Epidemiol. 2024 Jan 2;3(1):e254. doi: 10.1017/ash.2023.531 (PMC10762638; doi:10.1017/ash.2023.531)

**Supplementary Tables and Figures**

Supplementary Table 1. Specimen sources

| **Culture Source** | **n** |
| --- | --- |
| Blood | 130 |
| Peritoneal fluid | 12 |
| Bone | 1 |
| Deep Tissue | 1 |
| Joint/Synovial | 1 |
| Liver | 2 |
| Ovary | 1 |
| Pleural fluid | 2 |
| *Other sterile site (free text)* | |
| Deep Tissue | 1 |
| Other fluid | 3 |

Supplementary Table 2. Indwelling devices

| **Invasive Devices** | **n** |
| --- | --- |
| Central line | 109 |
| Urinary Catheter | 80 |
| PEG | 53 |
| Tracheostomy | 51 |
| Endotracheal/Nasotracheal tube | 11 |
| Nephrostomy | 0 |
| Nasogastric tube | 25 |
| *Other devices (free text)* | |
| Abscess drain | 1 |
| Arterial line | 3 |
| Chest tube | 2 |
| Ileostomy with catheter | 1 |
| IR Drain | 1 |
| JP drain | 4 |

Supplementary Table 3. Definition of carbapenem resistant Enterobacterales for this study

| **Organism** | **Antibiotic Susceptibility Based on Minimum Inhibitory Concentration (MIC)** | |
| --- | --- | --- |
| *Escherichia coli* | ***Resistant to:*** | ***AND Resistant to:*** |
| *Klebsiella pneumoniae* | Imipenem (MIC>/= 4) or  Meropenem (MIC >/=4) or  Doripenem (MIC >/=4) or | Ceftazidime (MIC >/= 16) and |
| *Klebsiella oxytoca* |  | Ceftriaxone (MIC >/= 4) and |
| *Klebsiella aerogenes* |  | Cefotaxime (MIC >/=4) |
| *Enterobacter cloacae* |  |  |

Supplementary Figure 1. Georgia Emerging Infections Program catchment area comprised of health district 3

Supplementary Figure 2. Directed acyclic graph of risk factors for indwelling devices and 90-day mortality.


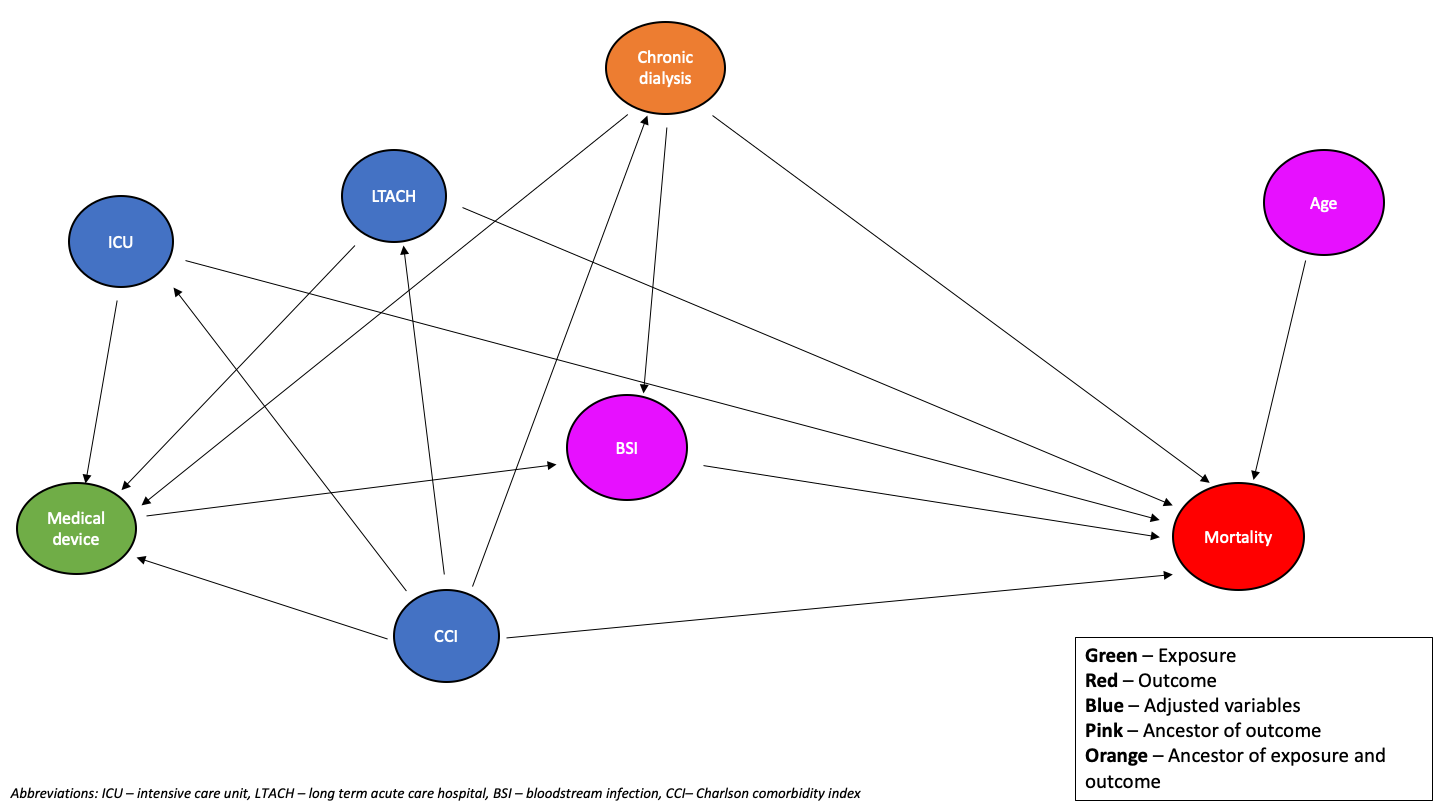

Supplement: Witt et al. supplementary material [file S2732494X23005314sup001.docx]
